# RSNA Expert Consensus Statement on Reporting Chest CT Findings Related to COVID-19: Interobserver Agreement Between Chest Radiologists

**DOI:** 10.1177/0846537120938328

**Published:** 2020-07-02

**Authors:** Danielle Byrne, Siobhan B. O’Neill, Nestor L. Müller, C. Isabela Silva Müller, John P. Walsh, Sabeena Jalal, William Parker, Ana-Maria Bilawichm, Savvas Nicolaou

**Affiliations:** 1 Department of Radiology, Vancouver General Hospital, British Columbia, Canada; 2 University of British Columbia, Vancouver, British Columbia, Canada; 3 Department of Radiology, Delfin Clinic, Salvador, Bahia, Brazil

**Keywords:** 2019n-CoV, COVID-19, CT, lung diseases, pneumonia

## Abstract

**Purpose:**

To assess the interobserver variability between chest radiologists in the interpretation of the Radiological Society of North America (RSNA) expert consensus statement reporting guidelines in patients with suspected coronavirus disease 2019 (COVID-19) pneumonia in a setting with limited reverse transcription polymerase chain reaction testing availability.

**Methods:**

Chest computed tomography (CT) studies in 303 consecutive patients with suspected COVID-19 were reviewed by 3 fellowship-trained chest radiologists. Cases were assigned an impression of typical, indeterminate, atypical, or negative for COVID-19 pneumonia according to the RSNA expert consensus statement reporting guidelines, and interobserver analysis was performed. Objective CT features associated with COVID-19 pneumonia and distribution of findings were recorded.

**Results:**

The Fleiss kappa for all observers was almost perfect for typical (0.815), atypical (0.806), and negative (0.962) COVID-19 appearances (*P* < .0001) and substantial (0.636) for indeterminate COVID-19 appearance (*P* < .0001). Using Cramer V analysis, there were very strong correlations between all radiologists’ interpretations, statistically significant for all (typical, indeterminate, atypical, and negative) COVID-19 appearances (*P* < .001). Objective CT imaging findings were recorded in similar percentages of typical cases by all observers.

**Conclusion:**

The RSNA expert consensus statement on reporting chest CT findings related to COVID-19 demonstrates substantial to almost perfect interobserver agreement among chest radiologists in a relatively large cohort of patients with clinically suspected COVID-19. It therefore serves as a reliable reference framework for radiologists to accurately communicate their level of suspicion based on the presence of evidence-based objective findings.

## Abstract (fr)

**Objectif:**

Évaluer la variabilité inter-observateurs entre radiologistes thoraciques concernant les lignes directrices de la déclaration de consensus d’experts de la Radiological Society of North America (RSNA) pour le signalement des patients ayant une pneumonie COVID-19 suspectée dans un établissement où la disponibilité des tests de dépistage par rt-PCR est limitée.

**Méthodes:**

Des études de tomodensitométries (TDM) thoraciques effectuées chez 303 patients consécutifs suspects de COVID-19 ont été analysées par 3 radiologistes thoraciques formés dans le cadre d’un fellowship. Chaque cas a été classé selon sa forme (typique, indéterminé, atypique ou négatif) pour la pneumonie COVID-19 en suivant les lignes directrices pour leur signalement tirées de la déclaration de consensus d’experts de la RSNA; une analyse inter-observateurs a ensuite été effectuée. Les caractéristiques objectives des TDM associées à la pneumonie COVID-19 et la répartition des constatations ont été consignées.

**Résultats:**

Le kappa de Fleiss pour tous les observateurs a été presque parfait pour toutes les formes typiques (0,815), atypiques (0,806) et négatives (0,962) de COVID-19 (*P* < 0,0001) et substantiel (0,636) pour les formes indéterminées de COVID-19 (*P* < 0,0001). L’analyse V de Cramer a montré de très fortes corrélations entre toutes les interprétations des radiologistes, avec une signification statistique pour toutes les formes (typiques, indéterminées, atypiques et négatives) de COVID-19 (*P* < 0,001). Les constatations objectives à l’imagerie par TDM ont été consignées avec des pourcentages semblables de cas typiques par tous les observateurs.

**Conclusion:**

La déclaration de consensus d’experts de la RSNA pour le signalement des constatations à la TDM thoracique en rapport avec la COVID-19 démontre une concordance substantielle à presque parfaite entre les radiologistes thoraciques dans une cohorte relativement importante de patients ayant une infection COVID-19 suspectée cliniquement. Il s’agit donc d’un cadre de référence fiable permettant aux radiologistes de communiquer avec exactitude leur niveau de soupçon en se basant sur la présence d’éléments objectifs reposant sur des données probantes.

## Introduction

Coronavirus disease 2019 (COVID-19) is a novel infectious disease caused by severe acute respiratory syndrome coronavirus 2. It was first identified in the human population in December 2019^1^ and spread rapidly around the world reaching pandemic status in March 2020.^2^ Reverse transcription polymerase chain reaction (RT-PCR) of respiratory specimens is the most widely used method for diagnosing COVID-19.^3,4^ However, in many clinical settings, RT-PCR testing may not be readily available, be limited to hospitalized patients, or may be initially falsely negative.^5^ Furthermore, patients with mild respiratory symptoms may be reluctant to travel to a PCR testing site or hospital.^5^

Although most radiological societies and professional organizations have recommended against performing chest computed tomography (CT) in the workup of patients with suspected COVID-19, in resource-poor countries where RT-PCR is not widely available, a tentative diagnosis or exclusion is often based on clinical and CT findings alone. Additionally, several retrospective studies have shown that CT has greater sensitivity (86%-98%) and lower false-negative rate than RT-PCR.^6-9^ The sensitivity of nasopharyngeal swabs ranges from 42% to 71%,^6,10^ with false negatives observed more frequently early in the course of the disease.^11,12^

This has led to the widespread use of chest CT in the workup of patients with suspected COVID-19 particularly in the setting of negative RT-PCR and high clinical suspicion.^6,9^ COVID-19 results in a spectrum of chest CT manifestations, which evolve over time ranging from peripheral predominant ground-glass opacities to an organizing pneumonia reaction pattern,^13^ with additional findings including crazy-paving and more diffuse ground glass, thus overlapping with imaging features of a variety of other disease processes including other infections, drug reaction, and inhalational exposure.^14-16^ As there are a number of etiologies with imaging findings that overlap with those of COVID-19, the inclusion of COVID-19 in the differential diagnosis of the radiology report may lead to unwarranted anxiety among referring physicians and patients. Optimal interpretation and reporting of the chest CT and clear, unambiguous communication with the referring physician are essential for optimal patient care as well as community and health care worker safety during the COVID-19 pandemic.

An expert consensus statement on reporting chest CT findings related to COVID-19, endorsed by the Society of Thoracic Radiology, the American College of Radiology, and the Radiological Society of North America (RSNA), was published to assist radiologists in recognizing and describing lung imaging findings in a standardized manner in patients under investigation for COVID-19 pneumonia and provide clarity in communication with other health care providers.^10^ The purpose of this study was to assess the interobserver variability between chest radiologists in the evaluation of CT scans in a cohort of patients with respiratory symptoms and suspected COVID-19 pneumonia using the RSNA expert consensus reporting guidelines in a setting with limited RT-PCR testing availability.

## Methods and Materials

### Study Population

Approval was obtained from the institutional review board, and the need for informed consent was waived for this retrospective study. A total of 303 consecutive patients (158 males and 145 females) with a median age of 49 years (range, 18-96) who presented with respiratory symptoms were suspected to have COVID-19 infection and underwent CT chest imaging between March 2, 2020, and March 16, 2020, were included. Due to the resource-limited nature of the hospital, reliable RT-PCR data were not available.

### Imaging Protocol

Chest CT studies were acquired on a 32-slice single-source CT scanner (Siemens Somatom Scope) using a standardized CT technique: 110 kVp, 345 mA max, pitch of 1.5, 0.6 seconds rotation time, and 1 mm scan thickness. Inspiratory phase CT chest examinations were acquired with patients in the supine position without administration of intravenous contrast.

### Imaging Analysis

Computed tomography images were extracted from the picture archiving and communication system, anonymized, and imported onto a secure browser-based viewing system for CT scans (MD.ai, available at https://www.md.ai/). The software displayed cross-sectional CT images only with soft tissue and lung kernel and allowed for dynamic scrolling, window width–window level adjustment, panning, and zoom. Three fellowship-trained chest radiologists, each with >10 years experience reading chest CT examinations (S.B.O.N., A.-M.B., and C.I.S.M.) performed diagnostic interpretation on all included CT studies. Observers, blinded to all patient information including RT-PCR result, recorded the presence or absence and distribution of typical COVID-19 imaging features (see Table 1) and scored each study according to the RSNA expert consensus reporting guidelines as to whether a study was typical, indeterminate, atypical, or negative for the presence of COVID-19 pneumonia.^10^

**Table 1.** Frequency Distribution of Typical COVID-19 Findings.

	Rad 1 (%)	Rad 2 (%)	Rad 3 (%)
Typical cases out of total (303)	140 (46.2%)	160 (52.8%)	139 (45.9%)
% of typical cases			
Ground glass total	137 (97.9%)	157 (98.1%)	130 (93.5%)
Round	124 (88.6%)	154 (96.3%)	124 (89.2%)
Peripheral	137 (97.9%)	156 (97.5%)	133 (95.7%)
Crazy paving total	55 (39.3%)	37 (23.1%)	44 (31.7%)
Round	31 (22.1%)	32 (20%)	40 (28.8%)
Peripheral	55 (39.3%)	40 (25%)	43 (30.9%)
Consolidation	91 (65%)	95 (59.4%)	72 (51.8%)
Round	49 (35%)	90 (56.3%)	61 (43.9%)
Peripheral	80 (57.1%)	104 (65%)	81 (58.3%)
Peribronchovascular	45 (32.1%)	43 (26.9%)	48 (34.5%)
Perilobular	70 (50%)	49 (30.6%)	29 (20.9%)
Consolidation with reverse halo	55 (39.3%)	20 (12.5%)	33 (23.7%)
Posterior distribution	140 (100%)	160 (100%)	139 (100%)
Bronchial dilatation	5 (3.6%)	11 (6.9%)	6 (4.3%)

### Statistical Analysis

Statistical analysis was performed using SPSS statistics version 25 (IBM). To quantify interobserver agreement, Cramer V and Fleiss kappa were determined across observers. Kappa values were obtained by reporting scores of each observer (according to the RSNA expert consensus statement on reporting chest CT findings related to COVID-19) to the median of the other 2 observers. Interobserver agreement was considered slight for a kappa value of 0.01 to 0.20, fair for 0.21 to 0.40, moderate for 0.41 to 0.60, substantial for 0.61 to 0.80, and almost perfect for 0.81 to 1.00.^17^ The result of a Cramer’s V-test lies between 0 and 1 and is interpreted as following: 0, no association; 0.05 to 0.1, weak; 0.1 to 0.15, moderate; 0.15 to 0.25, strong; >0.25, very strong.

## Results

**Figure 1.**
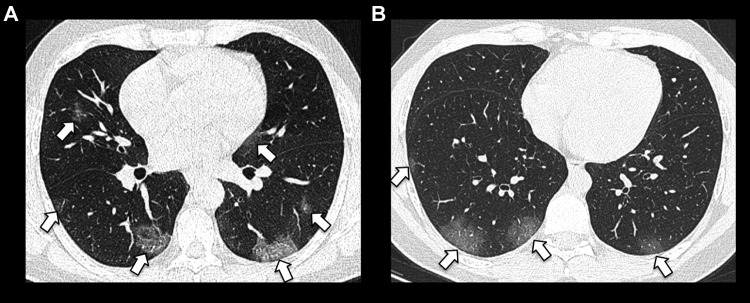
Bilateral posterior and peripheral predominant ground-glass opacities in (A) (short arrows), with a slightly more rounded appearance in (B) (short arrows).

**Figure 2. fig2-0846537120938328:**
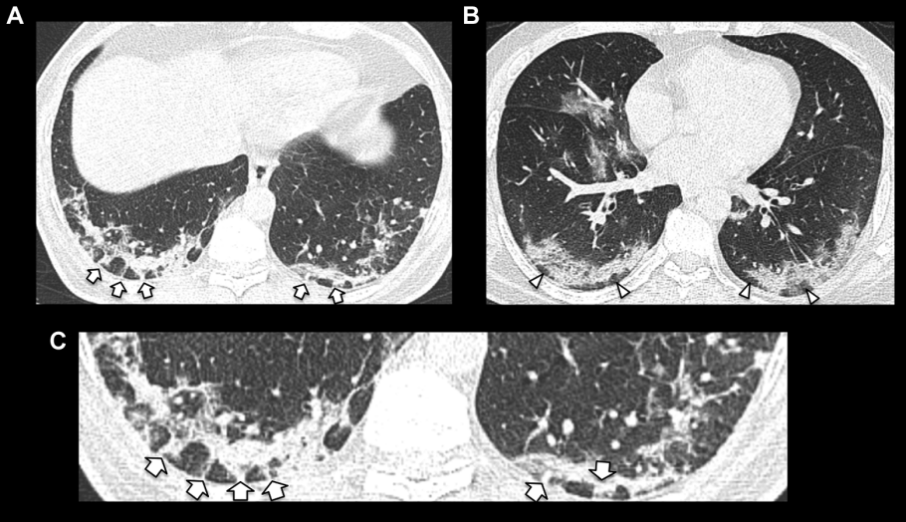
Examples of bilateral peripheral posterior predominant consolidation with perilobular morphology consistent with organizing pneumonia reaction pattern. Examples of perilobular arcades are demonstrated in (A) (short arrows) and areas of subpleural sparing in (B) (arrowheads). Magnified image of perilobular arcades demonstrated in (C).

**Figure 3. fig3-0846537120938328:**
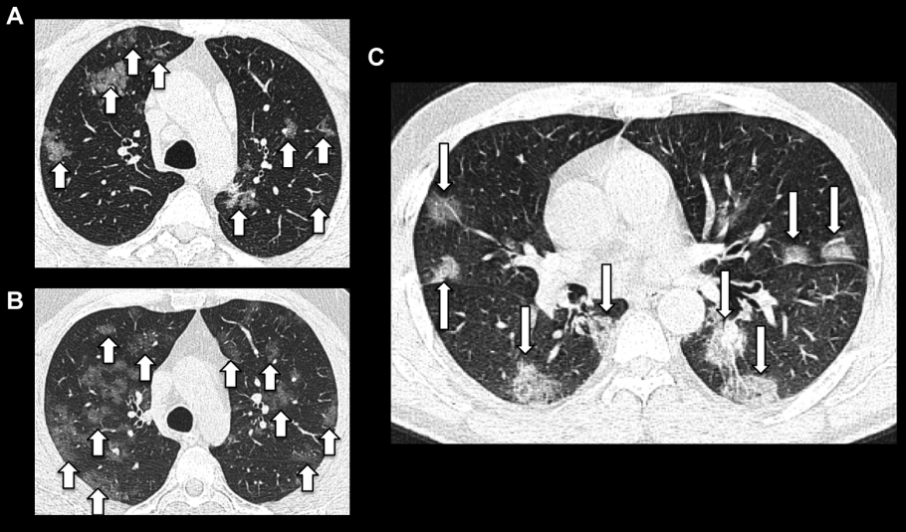
Examples of rounded peribronchovascular ground-glass opacities, slightly ill-defined in (A) and (B) (short arrows) and more confluent and well demarcated in (C) (long arrows).

**Figure 4. fig4-0846537120938328:**
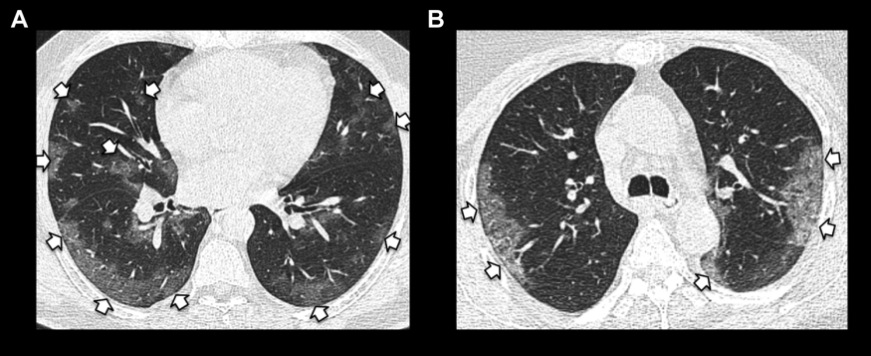
Spectrum of ground-glass opacities with peripheral predominance in (A) and (B) (short arrows).

**Figure 5. fig5-0846537120938328:**
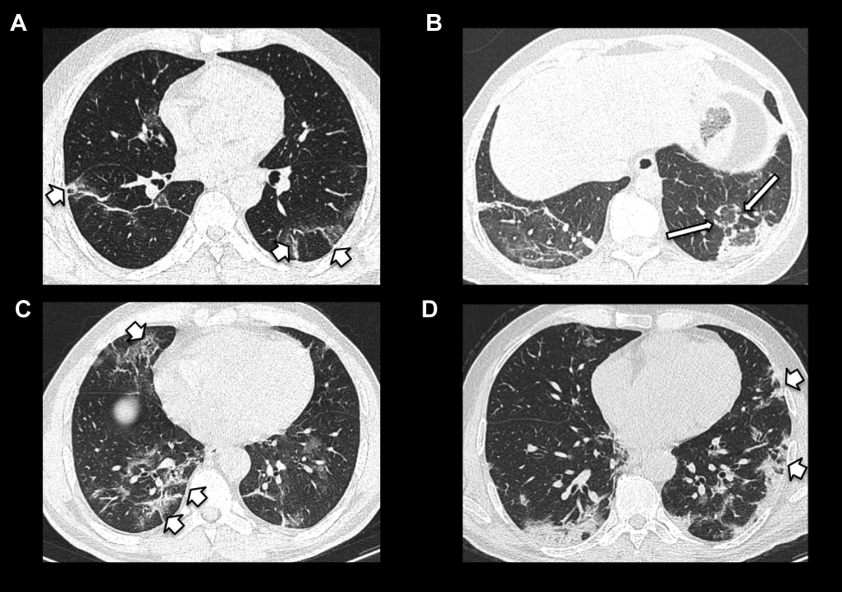
Spectrum of organizing pneumonia reaction pattern (short arrows) ranging from mild in (A), with peripheral crescentic, perilobular consolidation and central ground glass consistent with the “reverse halo” sign (long arrow) demonstrated in the left lower lobe in (B) and parenchymal distortion demonstrated in (C) and (D).

The Fleiss kappa for all observers was almost perfect (0.815) for typical COVID-19 appearance (*P* < .0001), substantial (0.636) for indeterminate COVID-19 appearance (*P* < .0001), and almost perfect for atypical (0.806) and negative (0.962) COVID-19 appearances (see Table 2). Using Cramer V analysis, there were very strong correlations between all radiologists’ interpretations, statistically significant for all (typical, indeterminate, atypical, and negative) COVID-19 appearances (*P* < .001; see Table 3). Of the cases with overall typical imaging appearance for COVID-19, specific imaging findings including ground glass, consolidation, and crazy-paving were recorded in similar percentages of cases by the observers (see Table 1). A posterior predominance of distribution was observed in all typical cases (100%). Examples of typical COVID-19 CT appearances are presented in Figures 1 [Fig fig2-0846537120938328] [Fig fig3-0846537120938328] [Fig fig4-0846537120938328] [Fig fig5-0846537120938328] to 6.

**Table 2.** Inter-Rater Agreement Between Chest Radiologists According to the RSNA Expert Consensus Statement on Reporting Chest CT Findings Related to COVID-19.

COVID-19 appearance	Agreement	Fleiss kappa	Standard error	*P* value	Interpretation
Typical COVID-19 appearance					
Radiologist 1 and radiologist 2 and radiologist 3		0.815		<.0001	Almost perfect agreement
Radiologist 1 and radiologist 2	90.1%	0.803	0.034	<.0001	Almost perfect agreement
Radiologist 1 and radiologist 3	91.1%	0.821	0.033	<.0001	Almost perfect agreement
Radiologist 2 and radiologist 3	91.1%	0.823	0.032	<.0001	Almost perfect agreement
Indeterminate COVID-19 appearance					
Radiologist 1 and radiologist 2 and radiologist 3		0.636		<.0001	Substantial agreement
Radiologist 1 and radiologist 2	89.1%	0.597	0.063	<.0001	Moderate agreement
Radiologist 1 and radiologist 3	89.4%	0.668	0.054	<.0001	Substantial agreement
Radiologist 2 and radiologist 3	89.8%	0.641	0.058	<.0001	Substantial agreement
Atypical COVID-19 appearance					
Radiologist 1 and radiologist 2 and radiologist 3		0.806		<.0001	Almost perfect agreement
Radiologist 1 and radiologist 2	98.0%	0.823	0.071	<.0001	Almost perfect agreement
Radiologist 1 and radiologist 3	98.3%	0.830	0.074	<.0001	Almost perfect agreement
Radiologist 2 and radiologist 3	97.7%	0.762	0.086	<.0001	Substantial agreement
Negative for pneumonia					
Radiologist 1 and radiologist 2 and radiologist 3		0.962		<.0001	Almost perfect agreement
Radiologist 1 and radiologist 2	98.3%	0.960	0.018	<.0001	Almost perfect agreement
Radiologist 1 and radiologist 3	98.7%	0.968	0.016	<.0001	Almost perfect agreement
Radiologist 2 and radiologist 3	98.3%	0.960	0.018	<.0001	Almost perfect agreement

Abbreviations: CT, computed tomography; RSNA, Radiological Society of North America.

**Table 3.** Correlation Between Chest Radiologists According to the RSNA Expert Consensus Statement on Reporting Chest CT Findings Related to COVID-19.

COVID-19 appearance	Cramer V	*P* value	Interpretation
Typical COVID-19 appearance			
Radiologist 1 and radiologist 2	0.810	<.001	Very strong correlation
Radiologist 1 and radiologist 3	0.821	<.001	Very strong correlation
Radiologist 2 and radiologist 3	0.831	<.001	Very strong correlation
Indeterminate COVID-19 appearance			
Radiologist 1 and radiologist 2	0.611	<.001	Very strong correlation
Radiologist 1 and radiologist 3	0.669	<.001	Very strong correlation
Radiologist 2 and radiologist 3	0.665	<.001	Very strong correlation
Atypical COVID-19 appearance			
Radiologist 1 and radiologist 2	0.823	<.001	Very strong correlation
Radiologist 1 and radiologist 3	0.842	<.001	Very strong correlation
Radiologist 2 and radiologist 3	0.774	<.001	Very strong correlation
Negative for pneumonia			
Radiologist 1 and radiologist 2	0.960	<.001	Very strong correlation
Radiologist 1 and radiologist 3	0.968	<.001	Very strong correlation
Radiologist 2 and radiologist 3	0.960	<.001	Very strong correlation

Abbreviations: CT, computed tomography; RSNA, Radiological Society of North America.

**Figure 6.**
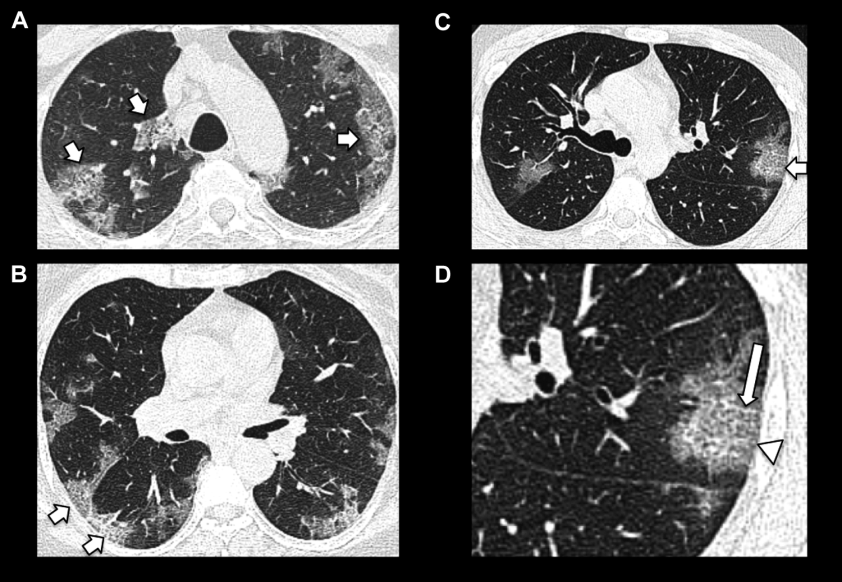
Examples of peripheral predominant ground-glass opacities with superimposed intralobular and interlobular septal thickening (short arrows) consistent with crazy paving (A-C). D, Magnified image of the left lung in (C) more clearly demonstrates intralobular and interlobular septal thickening (long arrow) superimposed on peripheral ground-glass opacification (arrowhead).

## Discussion

COVID-19 may result in a wide spectrum of CT imaging findings, which in isolation or combination can be of variable significance. Findings may vary depending on the severity of disease, time point in the infection, or the presence of background or coexistent lung disease. As a novel infection, by definition, experience of interpreting radiologists is limited, with continuously evolving described imaging features requiring time to establish diagnostic confidence. Given the potential implications of a COVID-19 diagnosis to the patient, exposed health care workers, and the wider community, concise and unambiguous radiology reporting is of paramount importance.

The RSNA expert consensus statement on reporting chest CT findings related to COVID-19 was published to facilitate reporting of CT imaging studies acquired during the COVID-19 pandemic.^10^ The statement’s main purpose was to provide radiologists with standardized reporting language to apply to patients under investigation for COVID-19 and to allow them to assign an evidence-based impression of their level of suspicion for COVID-19 infection, thus facilitating clear communication with referring physicians.^10^ Currently, there is a paucity of data on the initial experience utilizing the RSNA expert consensus statement guidelines in clinical practice.

Our results show that there is overall good agreement for typical, indeterminate, atypical, and negative imaging appearances according to the RSNA expert consensus statement reporting guidelines among expert chest radiologists in suspected COVID-19 cases where RT-PCR was largely unavailable. The RSNA expert consensus statement guidelines provide both standardized language and impression categories, which will likely reduce reporting variability and improve clarity in report interpretation among referring physicians. The CO-RADS (COVID-19 Reporting and Data System) is an additional recently described categorical reporting system to assess the degree of lung involvement by COVID-19 on chest CT in patients with moderate to severe symptoms with categories 1 to 5 according to increasing suspicion on CT.^18^ The CO-RADS system was not assessed in this study but reports very good performance in predicting COVID-19 patients with substantial interobserver agreement on initial clinical application.^18^

Commonly reported imaging features with greater specificity for COVID-19 pneumonia include peripheral and bilateral ground glass and/or multifocal ground glass with rounded morphology with or without consolidation or visible intralobular lines (“crazy paving”) and features of organizing pneumonia including the reverse halo sign.^10,13,19^ Ground-glass opacities and consolidation were recorded in a high percentage of typical COVID-19 cases among observers in our study with good agreement (see Table 1), emphasizing the importance of identifying these features on CT in patients with suspected COVID-19 where RT-PCR results may be delayed or unavailable. Posterior predominance of distribution^20^ has been reported in COVID-19 patients and was almost uniformly observed in the typical cases in this study. Bronchial dilatation in affected areas^21^ is less commonly seen in this study in line with recent literature.^22^ Computed tomography may be normal early on and subsequently depict an acute lung injury response to an infectious insult, with peripheral predominant multifocal ground glass with or without consolidation followed by organizing pneumonia reaction pattern.^23^

Due to the highly infectious nature of COVID-19 with estimated reproduction number (R0) of 2 to 2.53, rapid and accurate diagnostic methods are needed to identify, isolate, and treat the patients in a timely fashion, which could reduce mortality rates and the risk of public contamination. In cases where RT-PCR is initially negative, typical findings on CT may encourage maintenance of infection control mechanisms and prompt repeat testing.^8,24,25^ In cases of high clinical suspicion but negative RT-PCR, a combination of repeated swab tests and CT imaging may be useful to reach the diagnosis and assist management.^9,26^ With many countries beginning to ease lockdown restrictions and health care systems returning to near pre-pandemic levels of operation, it is important that radiologists remain vigilant to the typical COVID-19 CT imaging findings with the almost inevitable anticipated second wave of the pandemic. Such typical CT findings should be kept in mind for the foreseeable future, particularly in the outpatient CT reporting setting, as it is known that asymptomatic patients with COVID-19 can have abnormalities on CT.^27^

Our study has several limitations. Reverse transcription polymerase chain reaction was unavailable in the majority of cases and therefore we were only able to assess interobserver agreement according to the RSNA expert consensus statement guidelines in suspected COVID-19 cases. Furthermore, findings could not be correlated with timing of onset of infection, severity of symptoms, or patient outcome due to lack of available clinical data.

## Conclusion

The role of chest CT imaging in the era of COVID-19 continues to evolve with a particularly important role in cases with high clinical suspicion but negative or unavailable RT-PCR. We have shown that the RSNA consensus statement on reporting chest CT findings related to COVID-19 has good interobserver agreement among expert chest radiologists in a relatively large cohort of patients with suspected COVID-19 and unavailable RT-PCR testing. The guidelines provide a framework to which radiologists can easily refer and language which can easily be applied in daily clinical practice in order to accurately communicate their level of suspicion for COVID-19 based on the presence of evidence-based objective imaging findings.
